# Altered regulation of PDK4 expression promotes antiestrogen resistance in human breast cancer cells

**DOI:** 10.1186/s40064-015-1444-2

**Published:** 2015-11-10

**Authors:** William Walter, Jennifer Thomalla, Josh Bruhn, Dedra H. Fagan, Cheryl Zehowski, Douglas Yee, Andrew Skildum

**Affiliations:** Department of Biomedical Sciences, University of Minnesota Medical School, Duluth Campus, Duluth, MN USA; Masonic Cancer Center, University of Minnesota, Minneapolis, MN USA

**Keywords:** Breast cancer, Metabolism, Pyruvate dehydrogenase kinase-isoform 4 (PDK4), Antiestrogen resistance, Glucose

## Abstract

Acquired or de novo resistance to the selective estrogen receptor modulators tamoxifen and fulvestrant (ICI) is a major barrier to successful treatment of breast cancer. Gene expression patterns in tamoxifen resistant (TamR-MCF-7) cells were compared to their parental cells (MCF-7L) to identify an aberrantly regulated metabolic pathway. TamR-MCF-7 cells are cross resistant to ICI and doxorubicin, and have increased mitochondrial DNA. A small subset of genes had altered expression in TamR-MCF-7 relative to MCF-7L cells. One of the genes, pyruvate dehydrogenase kinase-4 (PDK4), phosphorylates pyruvate dehydrogenase (PDH). PDK4 expression was elevated in TamR-MCF-7 cells; this result was also observed in a second model of acquired antiestrogen resistance. PDK4 expression is controlled in part by glucocorticoid response elements in the PDK4 gene promoter. In MCF-7L cells, PDK4 mRNA expression was insensitive to glucocorticoid receptor agonists, while dexamethasone dramatically increased PDK4 expression in TamR-MCF-7 cells. Using siRNA to knock down PDK4 expression increased TamR-MCF-7 sensitivity to ICI; in contrast adapting cells to growth in glucose depleted media did not affect ICI sensitivity. Despite TamR-MCF-7 cells high levels of PDK4 mRNA relative to MCF-7L, TamR-MCF-7 cells have increased PDH activity. Wild type MCF-7 cells are reported to be heterozygous for a G to A mutation that results in a substitution of threonine for alanine near PDK4′s catalytic site. We found loss of heterozygosity in TamR-MCF-7 cells; TamR-MCF-7 are homozygous for the wild type allele. These data support a role for altered regulation of PDH by PDK4 and altered substrate utilization in the development of drug resistance in human breast cancer cells.

**Electronic supplementary material:**

The online version of this article (doi:10.1186/s40064-015-1444-2) contains supplementary material, which is available to authorized users.

## Background

Therapies that target the estrogen receptor-α (ER) have resulted in significant improvement in clinical outcomes for breast cancer patients (Early Breast Cancer Trialists’ Collaborative G 2005; Strasser-Weippl et al. 2013). These endocrine therapies include selective estrogen receptor modulators (SERMs) such as tamoxifen (Jordan and Koerner 1975), molecules that downregulate ER (e.g. fulvestrant Wakeling et al. 1991), and aromatase inhibitors that reduce expression of the endogenous ER ligand estradiol (Wells et al. 1978; Lipton et al. 1995). Despite these gains, recurrence of breast cancer after endocrine therapy is a major barrier to eliminating breast cancer mortality (Jordan and O’Malley 2007; Arpino et al. 2009; Moy et al. 2015). Mechanisms proposed for the development of resistance to ER targeted drugs include altered splicing or mutation of ER (Han et al. 2004; Scott et al. 1991; Banerji et al. 2012), reduced ER expression (Bayliss et al. 2007; Chu et al. 2007; Oh et al. 2001), aberrant tamoxifen metabolism (Osborne 1993; Osborne et al. 1992), altered function of cell cycle proteins regulating the G1 to S phase progression (Lehn et al. 2011; Abukhdeir et al. 2008; Varma et al. 2007; Mukherjee and Conrad 2005; Wilcken et al. 1997), altered interactions of scaffold proteins with downstream signaling proteins (Wallez et al. 2014; Brinkman et al. 2000), deregulated growth factor signaling (Fagan et al. 2012; Brockdorff et al. 2003), upregulated NF-kB signaling (Riggins et al. 2005), androgen receptor upregulation (Fujii et al. 2014), and activated Myc signaling (Mukherjee and Conrad 2005; Miller et al. 2011; Shajahan-Haq et al. 2014). Despite the many routes to endocrine therapy resistance in in vitro models, the clinical significance is less clear and has not led to predictive biomarkers or effective therapies to prevent or overcome resistance (Droog et al. 2013).

In the last two decades there has been a resurgence of interest in tumor metabolism as an exploitable target for cancer therapy, and metabolic abnormalities are now in the canon of hallmarks of cancer (Hanahan and Weinberg 2011; DeBerardinis et al. 2008). Cancers are generally more glycolytic than normal tissue, and this was originally interpreted as a compensatory response to defects in respiration (Cook and Higuchi 2012). One consequence of increased glycolysis is extracellular acidification, and this has been shown to cause insensitivity to endocrine therapies (Yang et al. 2013). Oncogenes and cell cycle regulatory proteins have been shown to regulate the metabolism of fuels in addition to cellular proliferation, with consequences for endocrine therapy in breast cancer cells (Shajahan-Haq et al. 2014; Lee et al. 2014; Wang et al. 2006). Cancer metabolism is diverse, and clinically the mitochondrial DNA (mtDNA) content of breast cancer varies with stage, with early and advanced stage cancers having higher mtDNA than those diagnosed at intermediate stage (Bai et al. 2011). This observation suggests that as cancers progress, they become more reliant on mitochondrial function. Consistent with that notion, malignant breast cancer cells are metabolically coupled with tumor associated fibroblasts, with fibroblasts fermenting substrates and providing lactate for oxidation by cancer cells; this relationship promotes SERM resistance (Martinez-Outschoorn et al. 2011). Taken together, these observations suggest that fuel metabolism is plastic in breast cancer cells, and that utilization of fuels impacts response to endocrine therapy.

A major determinant of fuel choice is the pyruvate dehydrogenase complex (PDH), an enzyme that converts pyruvate and coenzyme A to acetylCoA and carbon dioxide, reducing one NAD^+^ to NADH in the process. Different PDH subunits perform the decarboxylation, acyl transfer and oxidation/reduction reactions, and each subunit has cofactor requirements specific for their function. In addition to the abundance of substrate and product, PDH activity is also regulated by phosphorylation and dephosphorylation of the E1 subunit with inactivation of enzymatic activity by phosphorylation. The balance of kinase and phosphatase activities determines the PDH phosphorylation status. Four isoforms of pyruvate dehydrogenase kinase (PDK) are known, each active in response to different intracellular and extracellular conditions. PDK1 is activated by hypoxia (Mora et al. 2003; Kim et al. 2006); PDK2 is activated by the PDH products acetyl CoA and NADH and is inhibited by ADP and pyruvate (Hiromasa et al. 2006); and PDK3 is activated by ATP (Kato et al. 2005). Pyruvate dehydrogenase kinase-isoform 4 (PDK4), in contrast, is activated transcriptionally by hormonal signals such as retinoic acid and glucocorticoids, and transcriptionally repressed by insulin (Kwon et al. 2004). Thus while regulation of PDK1-3 reflects the immediate energy demands of the cell, PDK4 is reflective of whole organism energy balance and is upregulated in metabolic conditions such as hibernation (Buck et al. 2002), sustained exercise (Wang and Sahlin 2012; Pilegaard and Neufer 2004), and diabetes (Wu et al. 1998).

We previously reported that mitochondrial DNA copy number and mitochondrial superoxide were amplified in LCC9 cells, a cell line sequentially selected from parental MCF-7 cells for estrogen independence and fulvestrant resistance (Brunner et al. 1997; Skildum et al. 2011). We reasoned that mitochondrial amplification was a response to altered fuel metabolism, and that SERM resistant breast cancer cells would have altered expression of genes that regulate carbohydrate metabolism. In an independent model of acquired SERM resistance using cells selected directly from parental MCF-7 for growth in the presence of 4-hydroxytamoxifen (Fagan et al. 2012), we used quantitative PCR (qPCR) arrays to compare the expression of genes that regulate glucose metabolism. PDK4 expression was increased in tamoxifen resistant (TamR-MCF-7) cells, and the regulation of PDK4 by glucocorticoid receptor ligands was increased in TamR-MCF-7 cells. Paradoxically, PDH activity was also elevated in TamR cells. We found that parental MCF-7L cells were heterozygous for a mutation which causes an amino acid substitution near the PDK4 active site, and the TamR-MCF-7 cells lost heterozygosity and only expressed wild type PDK4. Decreased PDK4 expression partially restored antiestrogen sensitivity in these cells. We conclude that regulatory circuits that control oxidation of carbon derived from glucose are altered during selection for antiestrogen resistance.

## Methods

### Cell lines and reagents

The cell lines used are summarized in Table 1. ATCC-MCF-7 cells were obtained from the American Type Culture Collection (Soule et al. 1973). MCF-7L and TamR-MCF-7 cells were established as previously described (Fagan et al. 2012). LCC-MCF-7 and LCC9 cells were obtained from Dr. Robert Clarke of the Georgetown University Lombardi Cancer Center. ATCC-MCF-7, LCC-MCF-7 & MCF-7L were routinely cultured in phenol red free IMEM with 5 % fetal bovine serum (FBS), 1× penicillin/streptomycin, 6 ng/μL bovine pancreatic insulin (Sigma), and 2.5 μg plasmocin (Invivogen). TamR-MCF-7 cells were cultured in phenol red free IMEM + 5 % charcoal stripped FBS, 1× penicillin/streptomycin, 6 ng/μL insulin, 2.5 μg/L plasmocin and 10^−7^ M 4-hydroxytamoxifen (Sigma). LCC9 cells were cultured in phenol red free IMEM + 5 % charcoal stripped FBS, 1× penicillin/streptomycin, 6 ng/μL insulin, 2.5 μg/L plasmocin (Invivogen), and 10^−9^ M fulvestrant (ICI).

**Table 1:** Cell lines

Cell line name	Parental cell line	Selection for…	Source of cells	References
MCF-7L			University of Minnesota Masonic Cancer Center	
TamR-MCF-7	MCF-7L	Proliferation in the presence of 4-hydroxytamoxifen in vitro	University of Minnesota Masonic Cancer Center	Wallez et al. (2014)
LCC-MCF-7			Georgetown University Lombardi Cancer Center	
LCC9	LCC-MCF-7	Estrogen independent tumorigenesis in vivo; Proliferation in the presence of ICI in vitro	Georgetown University Lombardi Cancer Center	Pilegaard and Neufer (2004)
ATCC-MCF-7			American Type Culture Collection	Brunner et al. (1997)
MCF-7-GAL	ATCC-MCF-7	Proliferation in 4.5 mM galactose with undetectable glucose	Generated at University of Minnesota Medical School, Duluth	

The origins of the cell lines used in this study are summarized

MCF-7-GAL cells were generated by adapting ATCC-MCF-7 to progressively increased concentrations of galactose and decreased concentrations of glucose. Initially cells were cultured in DMEM (Sigma catalog number D5030) supplemented with 2 mM glutamine, 1 mM pyruvate, 5 % FBS, 1× penicillin/streptomycin, 12.5 mM d-glucose and 12.5 mM d-galactose; the concentration of glucose in this media is half that in MCF-7′s normal media. Over the course of 2 months, cells were allowed to grow to confluence, then split into new plates with progressively lower concentration of glucose and higher concentrations of galactose, until a population of cells was derived (MCF-7-GAL) that grew as rapidly in 25 mM galactose media as their parental cells grew in 25 mM glucose. In the 25 mM galactose media, some glucose was provided by FBS, however, the glucose concentration in this media was below the detection limit of a glucose oxidase based colorimetric assay (data not shown).

d-glucose, d-galactose, dexamethsone, mifepristone, 4-hydroxytamoxifen, and ICI were purchased from Sigma.

### Western blots

**Fig. 1:**
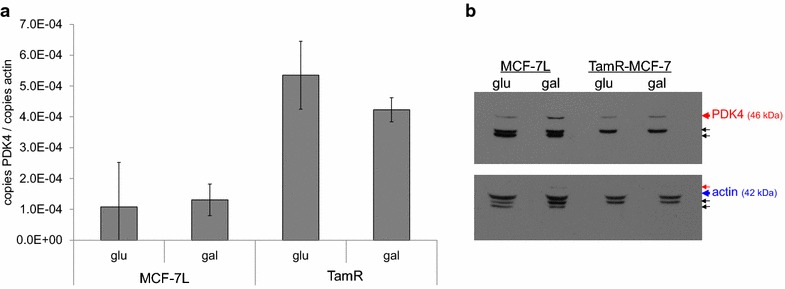
Glucose deprivation results in increased PDK4 protein without a change in mRNA. MCF-7L and TamR-MCF-7 cells were treated for 2 days in media containing 4.5 mM glucose or 4.5 mM galactose. **a** Total RNA was extracted, reverse transcribed, and the products used as template for qPCR amplification of PDK4 and b-actin. The average ratio is shown; *error bars* indicate one standard deviation (n = 3 biological replicates). **b** Whole cell lysates were prepared, and PDK4 and actin protein were visualized by western blotting, first for PDK4 (*upper panel*), then for actin (*lower panel*). The PDK4 band is indicated by a *red arrow*; background bands are indicated by *black arrows*. The actin band is indicated by a *blue arrow*

MCF-7L and TamR-MCF-7 were treated as described, and whole cell lysates were prepared in RIPA buffer supplemented with protease inhibitor cocktail, phenylmethanesulfonylfluoride, and sodium fluoride (all purchased from Sigma). Lysates were sonicated and cleared by high speed centrifugation. Protein concentration in the supernatants was determined by Bradford assay (BioRad), and equal quantities of protein were diluted in 6x-SDS-PAGE buffer and boiled. Lysate equivalent to 40 μg protein were resolved on 10 % SDS polyacrylamide gel (TGX gels, BioRad), then transferred to polyvinylidene fluoride membrane. The membrane was blocked with 5 % nonfat dry milk in phosphate buffered saline with 1 % Tween-20 (PBS-T; Tween-20 purchased from Sigma). Membranes were incubated overnight at 4 degrees with anti-PDK4 antibody (Abgent Cat. No. AP7041B) diluted at 1:1000 in blocking solution. The membrane was washed thrice with PBS-T, then incubated for 1 h with horseradish peroxidase conjugated secondary antibody diluted 1:1000 in blocking solution. The membrane was washed thrice with PBS-T, then incubated with enhanced chemiluminescence reagents for 2 min (SuperSignal West Pico; Pierce). Antibody interactions were visualized by exposing the membrane to film. The membrane was then washed with PBS-T and, using the procedure described above, probed for actin to ensure equal loading (Sigma clone AC-40). The images in Fig. 1b were generated by scanning films with a flatbed scanner.

### Cell cycle assay

MCF-7L and TamR-MCF-7 cells were plated at 10^6^ per 10 cm dish in MCF-7L’s normal growth media (permissive media for both cell lines). After attaching overnight, 1 nm ICI or vehicle (DMSO) was added to the cells without changing media. After 48 h, cells were harvested by trypsinization and fixed in ethanol. Cells were then centrifuged and the fixative removed by aspiration. The cell pellet was resuspended in 0.4 mL fluorescence-activated cell sorting buffer (1.37 M NaCl, 27 mM KCl, 43 mM Na2HPO4, 14.7 mM KH2PO4, 1 mg/mL ribonuclease A, 0.5 mM EDTA, 0.1 % Triton X-100, and 0.2 mg/mL propidium iodide), pipetted several times to ensure a uniform single-cell suspension, and transferred to a polystyrene tube. Cells were incubated at 4 °C in the dark for 30 min before analysis.

Cells were analyzed on a FACS Caliber flow cytometer, and data were collected using CellQuest Pro software (Becton–Dickinson). Cells were gated on forward and side scatter to eliminate debris and on the width versus area of the red fluorescent voltage pulse to eliminate cell aggregates. The area of the red fluorescence voltage pulse for the gated cells is proportional to their DNA content, and the cell cycle profile for each sample was estimated using ModFit LT software (Verity Software House). A minimum of 10,000 gated cells were analyzed for each sample, and triplicate parallel cultures were analyzed for each treatment.

### siRNA transfection and sulforhodamine B assays

TamR-MCF-7 cells were plated at 10,000 cells per well in 24 well plates in MCF-7L cells’ normal growth media. After overnight attachment, cells were transfected with one of two PDK4 siRNAs (Qiagen Flexitube Hs_PDK4_6 and Hs_PDK4_7) or a non-specific control siRNA using Lipofectamine 2000 (Invitrogen) according to the vendor’s protocol.

To validate efficacy of PDK4 knockdown, cells were treated with 1 nM dexamethasone or ethanol vehicle 1 day after transfection. Two days after treatment, total RNA was collected, reverse transcribed to cDNA, and used as template for qPCR amplification of PDK4 and actin using the methods described above.

One day after transfection cells were treated with 1 and 5 nM ICI or DMSO vehicle to test the effects of PDK4 knockdown on antiestrogen sensitivity. Four days later, cells were washed with PBS, then fixed in 10 % trichloroacetic acid for 30 min at 4 °C. The fixative was removed by aspiration and washing gently with tap water; the 24-well plates were then air dried. Each well was then stained with 0.4 % sulforhodamine B (SRB) in 1 % acetic acid at room temperature for 30 min, then washed thrice in 1 % acetic acid, and air dried. Stained proteins were then solubilized in 0.5 mL 10 mM tris base. A 0.1 mL aliquot was then transferred to a 96-well microtitre plate. SRB absorbance at 565 nm and background absorbance at 690 nm were measured spectrophotometrically.

To measure doxorubicin sensitivity, cells were plated at 5000 cells per well in 24 well plates and allowed to attach overnight. Cells were then treated ±0.02 micromolar doxorubicin (Sigma) from freshly prepared stocks diluted in water. Cells were cultured for 4 days, and cellular abundance was measured using SRB staining as described above.

### Quantitative PCR arrays

To compare gene expression patterns in parental MCF-7L cells and TamR-MCF-7 cells selected for antiestrogen resistance, we employed qPCR arrays containing primer sets targeting 81 genes that encode enzymes that control different aspects of carbohydrate metabolism (RT2 Profiler PCR Array Human Glucose Metabolism, Qiagen). Samples of MCF-7L and TamR-MCF-7 cells were generated by thawing ampules of each cell line and growing to sub-confluence in T75 flasks. Cells were then trypsinized and plated at 500,000 cells per 10 cm dish and allowed to attach overnight. Cells were then cultured for 2 days. Total RNA was isolated from cells using the Qiagen RNeasy kit. Using this protocol, four independent samples from each cell line were prepared on different days to account for biological variability in gene expression. Three sample sets were used for qPCR arrays, while the fourth set was used as a validation set.

RNA quantity and quality were measured using an Agilent Bioanalyzer, a core service provided by the University of Minnesota Genomics Center. The RNA integrity number (RIN) scores ranged from 8.60 to 9.10, indicating good quality of the RNA preparations. RNA was reverse transcribed and genomic DNA eliminated using the RT2 First Strand kit (Qiagen) according to the manufacturer’s instructions, and the products were used as template for qPCR arrays. The qPCR array plates were run on a Light Cycler 480 thermocycler (Roche), and data were analyzed using Microsoft Excel templates supplied by Qiagen.

### Pyruvate dehydrogenase activity assays

To measure PDH activity the Pyruvate Dehydrogenase Activity Colorimetric Assay Kit from BioVision was used. On three consecutive days, identically prepared aliquots of cells were thawed from liquid nitrogen and plated on T75 tissue culture plates. Cells were grown to sub-confluence, then split into 10 cm tissue culture dishes and grown for 2 days.

Assays were conducted on three consecutive days. On the day of each assay, cells were harvested by scraping cells and counted on a hemocytometer. 1x10^6^ cells were Dounce homogenized with 100 μL ice cold PDH assay buffer. The samples were kept on ice for 10 min before being centrifuged at 10,000×*g* for 5 min. The supernatant was then transferred to a fresh tube. Protein concentration in each sample was determined with the BioVision BCA Protein Assay Kit II using bovine serum albumin standards according to the manufacturer’s protocols. Equal amounts of protein were added from each sample and the reaction volume was adjusted to 50 μL with PDH Assay Buffer and transferred to a microtitre plate. Triplicate assays were performed on each sample, and blank reactions were included as negative controls. The plate was immediately measured with the plate reader in kinetic mode at 450 nm for 30 min at 37 °C. Separately, an external NADH standard curve was prepared using the BioVision protocol where 0, 2.5, 5.0, 7.5, 10 and 12.5 nmol/well of NADH standard was adjusted to 50 μL/well with PDH assay buffer. 50 μL of the reaction mix was added and immediately measured with the plate reader in equilibrium mode at 450 nm at 37 °C. The slopes of the kinetic measurements were used to calculate a rate of NADH produced per minute per microgram of protein.

### mtDNA copy number assay

To measure mitochondrial genome abundance relative to the nuclear genome, MCF-7L and TamR-MCF-7 cells were plated at 500,000 cells per 10 cm tissue culture dish, allowed to attach overnight, washed with PBS, and then treated with 10 nM 4-hydroxytamoxifen or ethanol vehicle for 4 days in phenol red free IMEM with 5 % steroid hormone depleted serum. Cells were then harvested by scraping after exposure to lysis buffer, and total RNase treated DNA was isolated using the Qiagen DNeasy kit according to the manufacturer’s instructions. 1 μg of DNA was used as template for qPCR amplification of mitochondrial DNA (a 125 bp portion of the cytochrome B coding sequence) and nuclear DNA (a 158 bp intron/exon boundary spanning fragment of the pyruvate kinase gene) as described above.

### Statistical analysis

Pairwise comparisons were subjected to Student T-tests using Microsoft Excel. Significant differences in experiments with multiple comparisons were evaluated using analysis of variance (ANOVA) followed by Tukey–Kramer Honest Significant Difference tests; this analysis was performed using JMP Pro Version 11.

## Results

**Fig. 2:**
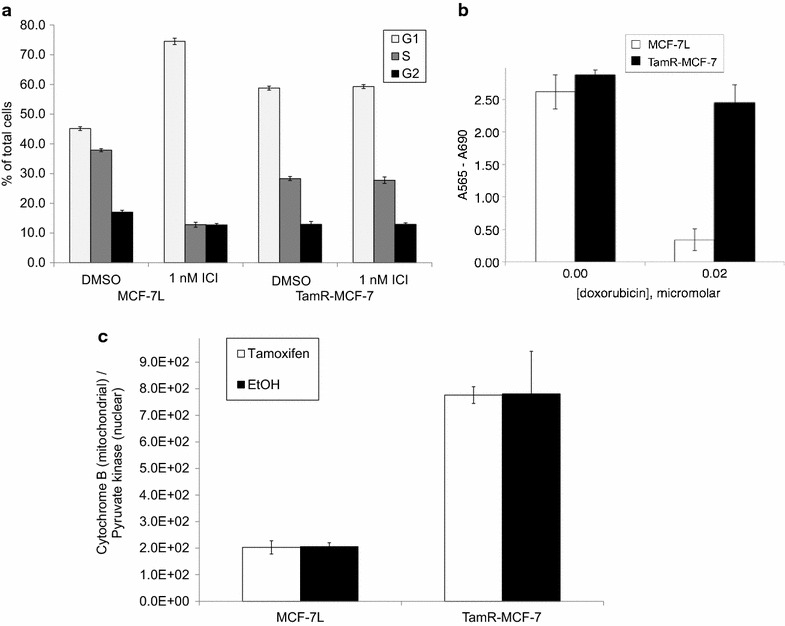
Tamoxifen resistant breast cancer cells have elevated mitochondrial DNA and cross resistance to ICI and doxorubicin. **a** MCF-7L and TamR-MCF-7 cells were treated for 2 days in 1 nM ICI or vehicle control (DMSO), then harvested and fixed in ethanol. Cell cycle phase distribution was determined by flow cytometric measurement of propidium iodide staining per cell. The average percentage of G1, S and G2/M cells are shown; *error bars* indicate one standard deviation (n = 3). **b** MCF-7L and TamR-MCF-7 cells were treated ±0.02 mm doxorubicin. After 4 days, total cellular mass was measured by sulforhodamine B staining. The average SRB staining is shown; *error bars* indicate one standard deviation (n = 6). **c** MCF-7L and TamR-MCF-7 cells were treated for 2 days with 10 nm 4-hydroxytamoxifen or control (EtOH). Genomic DNA was extracted and used as template for detection of cytochrome B DNA on the mitochondrial genome and pyruvate kinase DNA on the nuclear genome. The average ratio of cytochrome B to pyruvate kinase is shown; *error bars* indicate one standard deviation (n = 3)

To determine whether cells selected for resistance to the SERM 4-hydroxytamoxifen (TamR-MCF-7) had cross resistance to the pure antiestrogen fulvestrant (ICI), cell cycle phase distribution was compared in TamR-MCF-7 and parental cells (MCF-7L) after treatment with vehicle or 1 nM (ICI) in the presence of 5 % fetal bovine serum. Vehicle treated TamR-MCF-7 cells had an increased G1 phase population and decreased S phase population compared with vehicle treated MCF-7L cells, reflecting a slower rate of proliferation. While MCF-7L cells had an increased fraction of cells in the G1 phase and a decreased fraction in S phase, TamR-MCF-7 cell cycle phase distribution was unaffected by treatment with ICI (Fig. 2a), indicating cross resistance to ICI developed with selection for 4-hyroxytamoxifen resistance. Because resistance to antiestrogen has been associated with resistance to chemotherapy agents (Skildum et al. 2011), we then compared sensitivity to doxorubicin by measuring cell mass after treatment. While 0.02 μM doxorubicin resulted in near complete cytotoxicity of MCF-7L cells, TamR-MCF-7 cell growth was not affected (Fig. 2b).

To test whether resistance to endocrine and chemotherapy may result from or cause a change in metabolic capacity, we next compared mitochondrial gene dose by quantitative PCR (qPCR) measurement of pyruvate kinase, encoded in nuclear DNA, and cytochrome B, encoded in mitochondrial DNA (mtDNA) (Fig. 2c). We found that TamR-MCF-7 cells had threefold higher mtDNA copy number than parental MCF-7L cells. The mtDNA copy number was not altered by short term treatment with 4-hydroxytamoxifen in either cell line.

The elevated mtDNA copy number of TamR-MCF-7 cells suggests that a metabolic abnormality may be associated with the multi drug resistant phenotype in this model. To identify potential mediators of altered metabolism, expression of a panel of 81 genes that encode enzymes involved in carbohydrate metabolism was compared. MCF-7L and TamR-MCF-7 cells were serum starved, then treated with 1 nM 17β-estradiol for 1 day. Total RNA was isolated and reverse transcribed to cDNA, which was used as template in pathway targeted qPCR arrays. The expression of three genes exceeded a two-fold difference with statistical significance (*p* < 0.05) in TamR-MCF-7 cells: Glucokinase was expressed at lower levels in TamR-MCF-7, while phosphoglucomutase and PDK4 were expressed at higher levels in TamR-MCF-7 cells (Additional file 1: Figure S1). PDK4 encodes a regulatory kinase that phosphorylates and inhibits pyruvate dehydrogenase (PDH), a major determinant of substrate utilization in cells, and is the subject of the current study; the significance of glucokinase and phosphoglucomutase expression in this model will be explored separately.

**Fig. 3:**
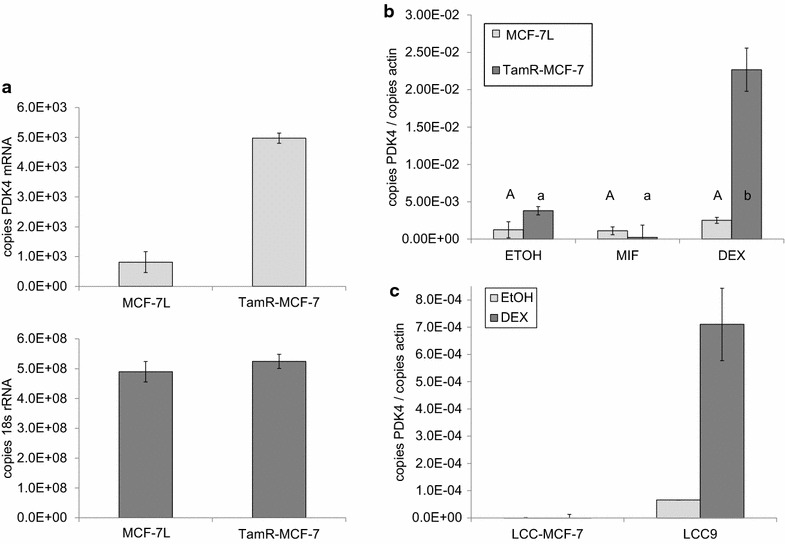
Tamoxifen resistant breast cancer cells have increased PDK4 mRNA expression. **a** cDNA prepared from cells treated identically to the cells used for the qCR arrays was used as template to measure expression of PDK4 mRNA and 18 s rRNA. Each reaction was performed in triplicate, and the average number of copies of PDK4 mRNA (*upper panel*) and 18s rRNA (*lower panel*) are shown; *error bars* indicate one standard deviation (n = 3 instrument replicates). **b** Triplicate cultures of MCF-7L and TamR-MCF-7 cells were treated with 1 nM mifepristone, 1 nM dexamethasone, or vehicle control (EtOH) for 2 days in serum free media. Total RNA was extracted, and PDK4 and actin expression were measured by qPCR. The average ratio of PDK4 and actin are shown; *error bars* indicate one standard deviation (n = 3 biological replicates). Statistically difference between the average ratios are indicated by *different letters* below the chart. **c** PDK4 and actin mRNA were measured by qPCR in triplicate cultures of LCC-MCF-7 and LCC9 cells cultured and treated as described in (**b**)

To confirm the expression difference in PDK4, primers were designed to amplify an exon boundary spanning fragment of its cDNA (Table 2). PDK4 was measured in cDNA from cells treated identically to those used in the qPCR arrays (the ‘validation set’) using qPCR; 18 s rRNA was used to confirm equal loading. While the pathway targeted qPCR arrays revealed a two fold relative difference in PDK4, using single gene qPCR with quantitation based on a five point standard curve, we found that PDK4 mRNA was five times more abundant in TamR-MCF-7 relative to their parental cells, with no difference in 18s rRNA (Fig. 3a).

**Table 2:** Primers

Primer name	Length (BP)	Forward primer	Reverse primer
18 s rRNA for qPCR	149	5′ TCA ACT TTC GAT GGT AGT CGC CGT 3′	5′ TCC TTG GAT GTG GTA GCC GTT TCT 3′
β-actin mRNA for qPCR	149	5′ GCC GCC AGC TCA CCA TGG AT 3′	5′ CAC CAT CAC GCC CTG GTG CC 3′
PDK4 mRNA for qPCR	119	5′ CCT GTG AGA CTC GCC AAC A 3′	5′ TCC ACC AAA TCC ATC AGG CTC 3′
Cytochrome B genomic for qPCR	125	5′-TGA TAT TTC CTA TTC GCC TAC ACA-3′	5′-TGT TGT TTG GAT ATA TGG AGG ATG-3′
Pyruvate kinase genomic for qPCR	158	5′-GTC GAT CCA GGA GAA CAT ATC AT-3	5′-CTC CTA GTT TTC ACC CTC ATT TTC-3′
PDK4 genomic for sequencing	367	5′ CAC TGA GAA TGT GAC CCG CT 3′	5′ AGC CTT GTG TGA AGT AAC CTT AG 3′
PDK4 cDNA for sequencing	393	5′ ATC CTC CCG ACC CAA TTA GT 3′	5′ ACC CAC TGC TAC CAC ATC ACA 3′

Sequences of primers used to measure expression of human PDK4 mRNA, b-actin mRNA, 18s rRNA, pyruvate kinase from the nuclear genome and cytochrome B from the mitochondrial genom are shown along with sequencing primers for PDK4 using genomic DNA and cDNA templates

The PDK4 promoter contains multiple glucocorticoid response elements. To test whether PDK4 expression was dependent on glucocorticoid receptor (GR) activity, we treated MCF-7L and TamR-MCF-7 with the GR agonist dexamethasone and the antagonist mifepristone in serum free media. To account for biological variability, triplicate cultures were prepared. In the cells treated with vehicle control (EtOH), we found elevated expression of PDK4 in TamR-MCF-7 cells (Fig. 3b), confirming our previous observations. While the GR ligands had no effect on PDK4 expression in MCF-7L cells, dexamethasone treatment resulted in an approximately five fold increase in PDK4 mRNA in TamR-MCF-7 cells. Mifepristone treatment alone caused a decrease in PDK4 expression in TamR-MCF-7 cells, but the change was not statistically significant.

To test whether increased PDK4 mRNA expression is a general phenomenon or is specific to the TamR-MCF-7 model of acquired antiestrogen resistance, we tested the dexamethasone induction of PDK4 mRNA in an independent model. In contrast to TamR-MCF-7 cells direct selection for growth in the presence of 4-hydroxytamoxien in vitro, LCC9 cells were selected from MCF-7 cells first for estrogen independence in ovariectomized nude mice, then selected for ICI resistance in vitro (Brunner et al. 1997). Relative to their parental LCC-MCF-7 cells, LCC9 cells exhibited higher basal PDK4 mRNA expression; dexamethasone treatment resulted in a ~tenfold increase in PDK4 mRNA abundance when treated in serum free media (Fig. 3c).

PDK4 is known to inhibit PDH, resulting in decreased oxidation of glucose derived carbon in the tricarboxylic acid cycle. Physiologically this results in conservation of glucose as cells switch to oxidation of fatty acids and amino acids. We reasoned that by limiting the ability of cells to gain ATP through glycolysis by substituting galactose for glucose (Frey 1996), we could increase expression of PDK4 and test whether increased PDK4 expression resulted in altered sensitivity to SERMs and doxorubicin. MCF-7L and TamR-MCF-7 cells were cultured for 2 days in media containing fetal bovine serum and either 4.5 mg/mL glucose or 4.5 mg/mL galactose. PDK4 mRNA expression was measured by qPCR, and PDK4 protein was measured by western blot. PDK4 mRNA expression was not altered by limiting the availability of glucose in either cell line (Fig. 1a). In contrast to the consistently elevated PDK4 mRNA observed in TamR-MCF-7, we found no difference in PDK4 protein detected by Western blot (Fig. 1b); Western blotting was challenging due to the consistent presence of non-specific background bands (indicated by black arrows in Fig. 1b). PDK4 protein was increased by culturing cells in galactose in both cell lines, but with a greater magnitude of change in drug sensitive MCF-7L cells.

**Fig. 4:**
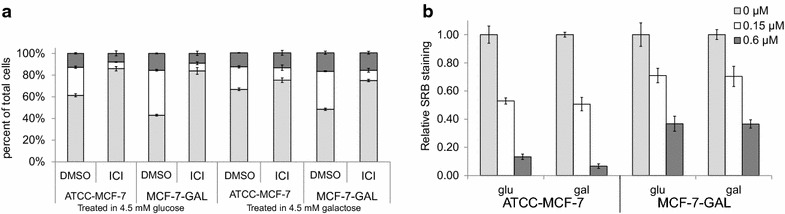
Adaptation to glucose deprivation increases resistance to doxorubicin without altering antiestrogen sensitivity. ATCC-MCF-7 cells were adapted to growth in glucose restricted media to generate MCF-7-GAL cells. **a** MCF-7-GAL and galactose naïve parental cells were treated with 1 nM ICI or control in media containing 4.5 mM glucose or 4.5 mM galactose. After 2 days, cell cycle phase distribution was determined by propidium iodide staining and flow cytometry. The average percent of cells in G1 phase (*light gray*), S phase (*white*) and G2/M phase (*dark gray*) are shown; *error bars* indicate one standard deviation (n = 3). **b** MCF-7-GAL and galactose naïve parental cells were treated with the indicated concentrations of doxorubicin for 24 h in media containing either 4.5 mM glucose or 4.5 mM galactose. Cellular mass was measured via sulforhodamine B (SRB) assay. The average SRB staining is shown relative to untreated cells; *error bars* indicate one standard deviation (n = 6)

To test the effects of carbohydrate substitution on drug sensitivity, ATCC-MCF-7 cells were adapted to growth in glucose limited conditions by culturing in progressively greater galactose and lower glucose concentrations until a population was derived that could grow as well in 4.5 mM galactose as parental cells grew in 4.5 mM glucose; these cells were designated MCF-7-GAL. To test whether ATP generated through glucose fermentation was required for sensitivity to antiestrogens, parental and MCF-7-GAL cells were treated with 1 nM ICI; treatments were performed in both glucose or galactose media. Cell cycle phase distribution was then determined by DNA staining and flow cytometry (Fig. 4a). We found that parental MCF-7 cells were sensitive to ICI in both media, showing an increase in G1 phase cells and a decrease in S phase cells, though the degree of G1 arrest was reduced when treated in galactose. MCF-7-GAL cells had a reduced G1 and increased S phase fractions in the absence of ICI. MCF-7-GAL cells exhibited a G1 arrest with ICI treatment in both media, suggesting that the fermentation of glucose is not a determinant of SERM sensitivity in this model.

In contrast, adaptation to galactose resulted in decreased sensitivity to doxorubicin. Parental MCF-7 and MCF-7-GAL cells were treated with doxorubicin for 2 days, after which cell mass was measured by SRB staining. Galactose adapted cells had a smaller decrease in cell mass after treatment with either 0.15 or 0.6 μM doxorubicin (Fig. 4b).

**Fig. 5:**
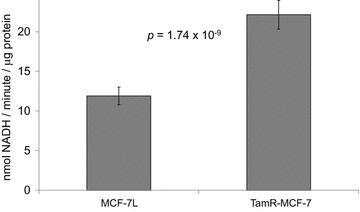
Tamoxifen resistant breast cancer cells have increased pyruvate dehydrogenase activity. On three consecutive days, lysates were prepared from identically treated MCF-7L and TamR-MCF-7 cells. Equal amounts of protein were subjected to a pyruvate dehydrogenase (PDH) assay. The average PDH activity expressed as NADH produced per minute per microgram of protein is shown for each assay replicate; *error bars* indicate one standard deviation (n = 3 biological replicates × 3 instrument replicates). The *p* value shown is from a two sided unpaired Student t test

Because drug resistant TamR-MCF-7 cells had increased PDK4 mRNA but not PDK4 protein, we next compared the activity of PDK4′s regulatory target, PDH. MCF-7L and TamR-MCF-7 cells were cultured in growth media, and lysates were prepared and subjected to an in vitro PDH activity assay that measured the rate of NADH generation. Cultures were prepared such that identical independent lysates could be prepared and the PDH assay conducted using fresh lysates on three consecutive days. Despite having greater expression of PDK4 mRNA, TamR-MCF-7 cells consistently had significantly elevated PDH activity compared to parental MCF-7L cells (Fig. 5; Additional file 1: Figure S2), suggesting TamR-MCF-7 cells have a greater capacity to oxidize glucose.

Because TamR-MCF-7 cells had, paradoxically, elevated PDK4 mRNA expression and elevated PDH activity relative to their parental cells, we wondered if the TamR cells expressed an altered form of PDK4. The Catalogue of Somatic Mutations In Cancer (COSMIC) database (Forbes et al. 2008, 2015) reports that MCF-7 cells are heterozygous for a point mutation that results in a non-conservative alanine to threonine substitution at amino acid position 144. This position is in alpha helix #7, near the PDK4 catalytic site (Wynn et al. 2008). We designed sequencing primers around this site (Table 2) and sequenced the region using cDNA and genomic DNA as template. We confirmed the heterozygous genotype reported in the COSMIC database in MCF-7L cells (Additional file 1: Figure S3). We found that TamR-MCF-7 cells had loss of heterozygosity at this position; surprisingly TamR-MCF-7 were homozygous for the wild type PDK4 sequence.

**Fig. 6:**
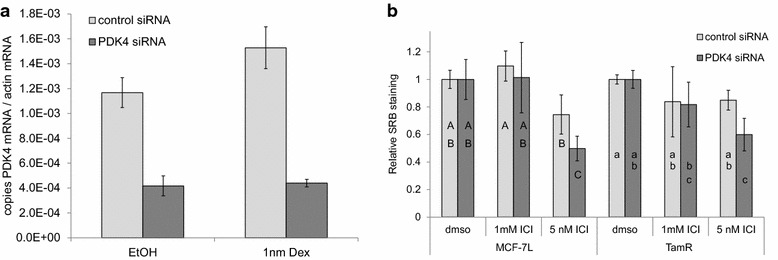
Reducing PDK4 expression partially restores sensitivity to antiestrogen in tamoxifen resistant breast cancer cells. **a** TamR-MCF-7 cells were transfected with siRNA targeted to PDK4 or a non-specific control siRNA. The next day, they were treated with 1 nm dexamethasone in media containing fetal bovine serum. Two days later, total RNA was collected, and PDK4 and β-actin mRNA expression was measured by qPCR as described. The average ratio of PDK4 mRNA to b-actin mRNA is shown; *error bars* indicate one standard deviation (n = 3 biological replicates). **b** MCF-7L and TamR-MCF-7 cells were transfected with PDK4 or control siRNA. The next day, cells were treated with the indicated doses of ICI or vehicle control. After 4 days, cellular mass was measured by sulforhodamine B staining. The average SRB staining is shown relative to vehicle treated samples; *error bars* indicate one standard deviation (n = 4). *Different letters* indicate statistically significant differences determined by ANOVA and Tukey–Kramer honest significant differences comparisons

To test the functional significance of PDK4 expression on antiestrogen sensitivity, we used silencing RNA (siRNA) to knock down PDK4 expression in TamR-MCF-7 cells. To validate the efficacy of the siRNA, TamR-MCF-7 cells were transfected with PDK4 or control siRNA, then treated with dexamethasone or vehicle control in media containing 5 % FBS. RNA was isolated and reverse transcribed to cDNA, and the products used as template for qPCR amplification of PDK4 and actin (Fig. 6a). In these conditions, dexamethasone treatment resulted in a smaller increase in PDK4 expression compared to treatment in serum free media (Fig. 2c).

TamR-MCF-7 cells were then transfected with PDK4 or control siRNA and treated with 1 or 5 nM ICI for 4 days, after which cellular abundance was measured by SRB staining. A small but reproducible and statistically significant increase in ICI sensitivity was observed with PDK4 siRNA relative to control siRNA at the highest 5 nm dose (Figs. 6b), suggesting PDK4 expression can mediate the responsiveness to SERMs. This observation was reproduced using a second siRNA targeting a different region of the PDK4 mRNA 3′ untranslated region (Additional file 1: Figure S4).

## Discussion

The data presented above point to altered regulation of pyruvate dehydrogenase as mediating breast cancer cell sensitivity to estrogen receptor ligands such as tamoxifen and ICI, and reveal an important role for the serine threonine kinase PDK4 in acquired SERM resistance. PDK4 is reported to phosphorylate and inhibit PDH, preventing the oxidation of carbon derived from glucose. When PDH is inactive, cells generate ATP through glycolysis, oxidation of amino acids and/or fatty acid oxidation. Rapidly dividing cells such as cancer cells require not only ATP but also carbon for biosynthesis, and the inhibition of PDH may allow glucose derived carbon to be conserved for biosynthesis of ribose sugars, amino acids, and glycerol, and the reduction of NADP^+^ to NADPH. We show that sensitivity to SERMs is not affected by replacing glucose with galactose, a non-fermentable substrate. In contrast, galactose adapted cells showed resistance to the chemotherapy agent doxorubicin; this phenomenon has been reported previously in liver cancer cells (Marroquin et al. 2007).

PDK4 expression is transcriptionally regulated by numerous promoter elements, and its elevated mRNA in TamR-MCF-7 cells could indicate disruption of several signal transduction pathways. In a glioblastoma, Kim et al. describe PDK4 regulation through NF-κB activation of PGC1α (Kim et al. 2015), and NF-κB signaling is altered in the LCC9 model of acquired SERM resistance (Riggins et al. 2005). Hypoxic signaling through estrogen related receptor gamma (ERRγ) also activates PDK4 transcription (Lee et al. 2012; Zhang et al. 2006), and ERRγ is directly inhibited by 4-hydroxytamoxifen, the most biologically potent tamoxifen metabolite (Coward et al. 2001; Tremblay et al. 2001). Knutson et al. report that PDK4 expression is dependent on progesterone receptor sumoylation in MCF-7 cells (Knutson et al. 2012), and progesterone receptor regulates breast cancer cell proliferation (Skildum et al. 2005). Mifepristone is an antagonist of both glucocorticoid receptors and progesterone receptors (Beck et al. 1993), though we did not observe statistically significant regulation of PDK4 mRNA by mifepristone in either MCF-7L or TamR-MCF-7 (Fig. 2b). Interestingly, TamR-MCF-7 express reduced progesterone receptor mRNA relative to their parental cells (Fagan et al. 2012).

PDK4 was first identified in a linkage study of loci associated with Type 2 diabetes mellitus in Native Americans (Rowles et al. 1996), and insulin is known to repress PDK4 transcription (Wu et al. 1998; Majer et al. 1998). Interestingly, in serum free media we show that the regulation of PDK4 by the glucocorticoid receptor agonist dexamethasone only occurs in drug resistant breast cancer cells (Fig. 2c), while the dexamethasone induction is dramatically blunted when TamR-MCF-7 cells are treated in the presence of fetal bovine serum (Fig. 7a; Additional file 1: Figure S4a). Insulin inhibits glucocorticoid dependent expression of PDK4 expression by preventing FOXO1 translocation to the nucleus (Kwon et al. 2004). Together these results suggest that in the absence of insulin and other growth factors in serum, parental MCF-7 cells have increased FOXO1 activity compared to TamR-MCF-7 cells, but that serum growth factors are still capable of blunting PDK4 expression by dexamethasone in these cells.

The consequences of PDK4 expression on cancer progression and response to therapy are likely to be context dependent. PDK4 was shown to be abundant in patient glioblastoma compared to normal tissue, and indirectly reducing its expression through shRNA targeting the RelA subunit of NF-kB resulted in reduced growth of xenografts in nude mice (Kim et al. 2015). Similarly, reducing PDK4 directly through shRNA resulted in increased tumor free survival of mice bearing prostate cancer cell xenografts (Liu et al. 2014). In contrast, in lung cancer cells decreasing PDK4 expression both prevented the antiproliferative action of a PPARγ agonist by blocking its action at the G1 → S phase checkpoint (Srivastava et al. 2014) and promoted epithelial to mesenchymal transition and chemotherapy resistance. The latter effects may be due to PDK4 physically interacting with the mitochondrial apoptosis inducing factor (AIF) rather than its phosphorylation of PDH (Sun et al. 2014; Cande et al. 2002).

Although PDK4 mRNA is overexpressed in TamR-MCF-7 cells relative to their tamoxifen naïve parental cells, PDK4 protein levels are similar and PDH activity is higher in TamR-MCF-7. These results suggest that PDK4 in TamR-MCF-7 is not acting as an inhibitor of PDH. Increased mRNA without increased protein level is consistent with decreased translation and/or increased protein turnover. If PDK4 protein turnover is increased in TamR-MCF-7 cells, the increased PDH activity in these cells may be result because PDK4 does not have time to be appropriately post-translationally modified or transported to the mitochondria. PDK4 has several sites identified as phosphorylated in proteomic screens (Franchin et al. 2015; Hornbeck et al. 2012), but the significance of these modifications with respect to PDK4 or PDH activity have not been characterized. Newly synthesized PDK4 may have reduced PDH inhibitory activity, and/or may act through targets other than PDH (e.g. AIF). These possibilities, and the significance of PDK4 polymorphisms, are currently under study.

## Additional file

10.1186/s40064-015-1444-2 **Figure S1.** Volcano plot showing glucose metabolism genes differently expressed in TamR-MCF-7 and parental MCF-7L. Expression of glucose metabolism genes were compared using qPCR arrays. Genes expressed higher in TamR-MCF-7 cells are represented by dots on the right side of the plot while genes expressed at lower levels in TamR-MCF-7 are on the left side. qPCR arrays were performed on separately prepared triplicate cultures; the vertical axis shows the p value for each gene. The dots representing phosphoglucomutase (PGM), glucokinase (GCK) and pyruvate dehydrogenase kinase -4 (PDK4) are indicated by gray circles. **Figure S2.** TamR-MCF-7 cells have increased pyruvate dehydrogenase activity relative to parental MCF-7L cells. On three consecutive days, lysates were prepared from identically treated MCF-7L and TamR-MCF-7 cells. Equal amounts of protein from each cell line were subjected to a pyruvate dehydrogenase (PDH) assay. The average PDH activity expressed as NADH produced per minute per microgram of protein is shown for each assay replicate; error bars indicate one standard deviation (n = 3 instrument replicates). Asterisks indicate significantly different (p < 0.05) averages as determined by unpaired two tailed Student’s T tests. The numbers below each day indicate the amount of protein loaded in each assay. **Figure S3.** Tamoxifen resistant breast cancer cells have loss of heterozygosity at amino acid position 144 in the PDK4 protein. MCF-7L cDNA and genomic DNA was used as template for sequencing of the DNA around base position 430 of PDK4 mRNA. Forward and reverse reads are shown; wild type and mutant sequences are shown below. **Figure S4.** Reducing PDK4 expression partially restores sensitivity to antiestrogen in tamoxifen resistant breast cancer cells. A) TamR-MCF-7 cells were transfected with an siRNA targeting a different region of the PDK4 mRNA than that shown in Fig. 6 or a non-specific control siRNA. The next day, they were treated with 1nm dexamethasone in media containing fetal bovine serum. Two days later, total RNA was collected, and PDK4 and β-actin mRNA expression was measured by qPCR as described. The average ratio of PDK4 mRNA to b-actin mRNA is shown; error bars indicate one standard deviation (n = 3 biological replicates). B) MCF-7L and TamR-MCF-7 cells were transfected with the PDK4 siRNA used in Figure S4A or control siRNA. The next day, cells were treated with the indicated doses of ICI or vehicle control. After four days, cellular mass was measured by sulforhodamine B staining. The average SRB staining is shown relative to vehicle treated samples; error bars indicate one standard deviation (n = 4). Different letters indicate statistically significant differences determined by ANOVA and Tukey-Kramer Honest Significant Differences comparisons.
