# Supplementary material for: Altered regulation of PDK4 expression promotes antiestrogen resistance in human breast cancer cells
Source: Springerplus. 2015 Nov 10;4:689. doi: 10.1186/s40064-015-1444-2 (PMC4641142; doi:10.1186/s40064-015-1444-2)
Supplement: Supplementary file 1 — 10.1186/s40064-015-1444-2 Figure S1. Volcano plot showing glucose metabolism genes differently expressed in TamR-MCF-7 and parental MCF-7L. Expression of glucose metabolism genes were compared using qPCR arrays. Genes expressed higher in TamR-MCF-7 cells are represented by dots on the right side of the plot while genes expressed at lower levels in TamR-MCF-7 are on the left side. qPCR arrays were performed on separately prepared triplicate cultures; the vertical axis shows the p value for each gene. The dots representing phosphoglucomutase (PGM), glucokinase (GCK) and pyruvate dehydrogenase kinase -4 (PDK4) are indicated by gray circles. Figure S2. TamR-MCF-7 cells have increased pyruvate dehydrogenase activity relative to parental MCF-7L cells. On three consecutive days, lysates were prepared from identically treated MCF-7L and TamR-MCF-7 cells. Equal amounts of protein from each cell line were subjected to a pyruvate dehydrogenase (PDH) assay. The average PDH activity expressed as NADH produced per minute per microgram of protein is shown for each assay replicate; error bars indicate one standard deviation (n = 3 instrument replicates). Asterisks indicate significantly different (p < 0.05) averages as determined by unpaired two tailed Student’s T tests. The numbers below each day indicate the amount of protein loaded in each assay. Figure S3. Tamoxifen resistant breast cancer cells have loss of heterozygosity at amino acid position 144 in the PDK4 protein. MCF-7L cDNA and genomic DNA was used as template for sequencing of the DNA around base position 430 of PDK4 mRNA. Forward and reverse reads are shown; wild type and mutant sequences are shown below. Figure S4. Reducing PDK4 expression partially restores sensitivity to antiestrogen in tamoxifen resistant breast cancer cells. A) TamR-MCF-7 cells were transfected with an siRNA targeting a different region of the PDK4 mRNA than that shown in Fig. 6 or a non-specific control siRNA. The next day, they were treated wit [file 40064_2015_1444_MOESM1_ESM.pptx]

## Slide 1
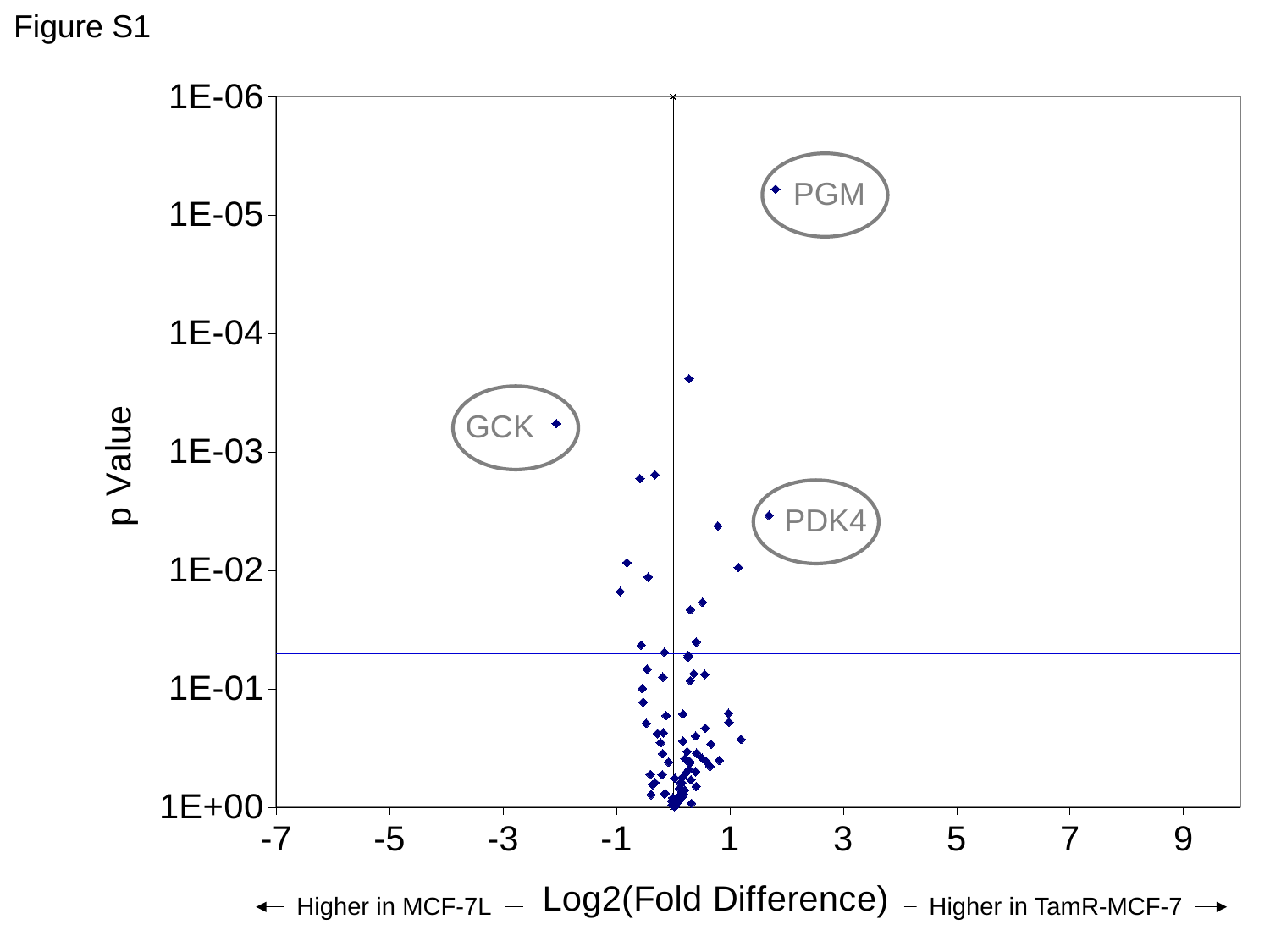

Figure S1
### Chart
| Category | | | |
|---|---|---|---|
PGM
GCK
PDK4
Higher in MCF-7L
Higher in TamR-MCF-7

## Slide 2
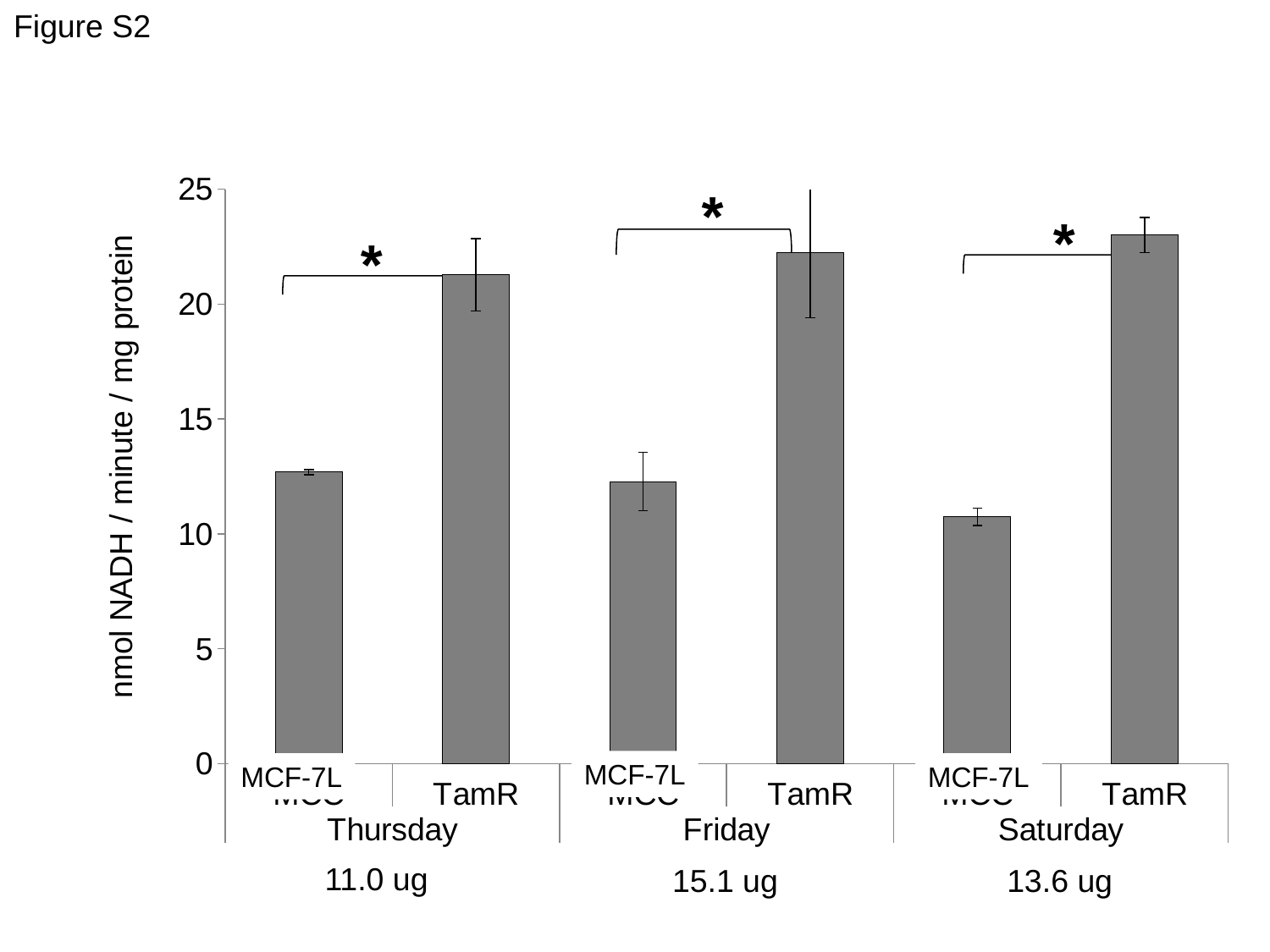

Figure S2
### Chart
| Category | |
|---|---|
| MCC | 12.69231473993594 |
| TamR | 21.27323699611832 |
| MCC | 12.27701133771977 |
| TamR | 22.22563075599338 |
| MCC | 10.73506852958643 |
| TamR | 23.00874554887968 |*
*
*
MCF-7L
MCF-7L
MCF-7L
11.0 ug
15.1 ug
13.6 ug

## Slide 3
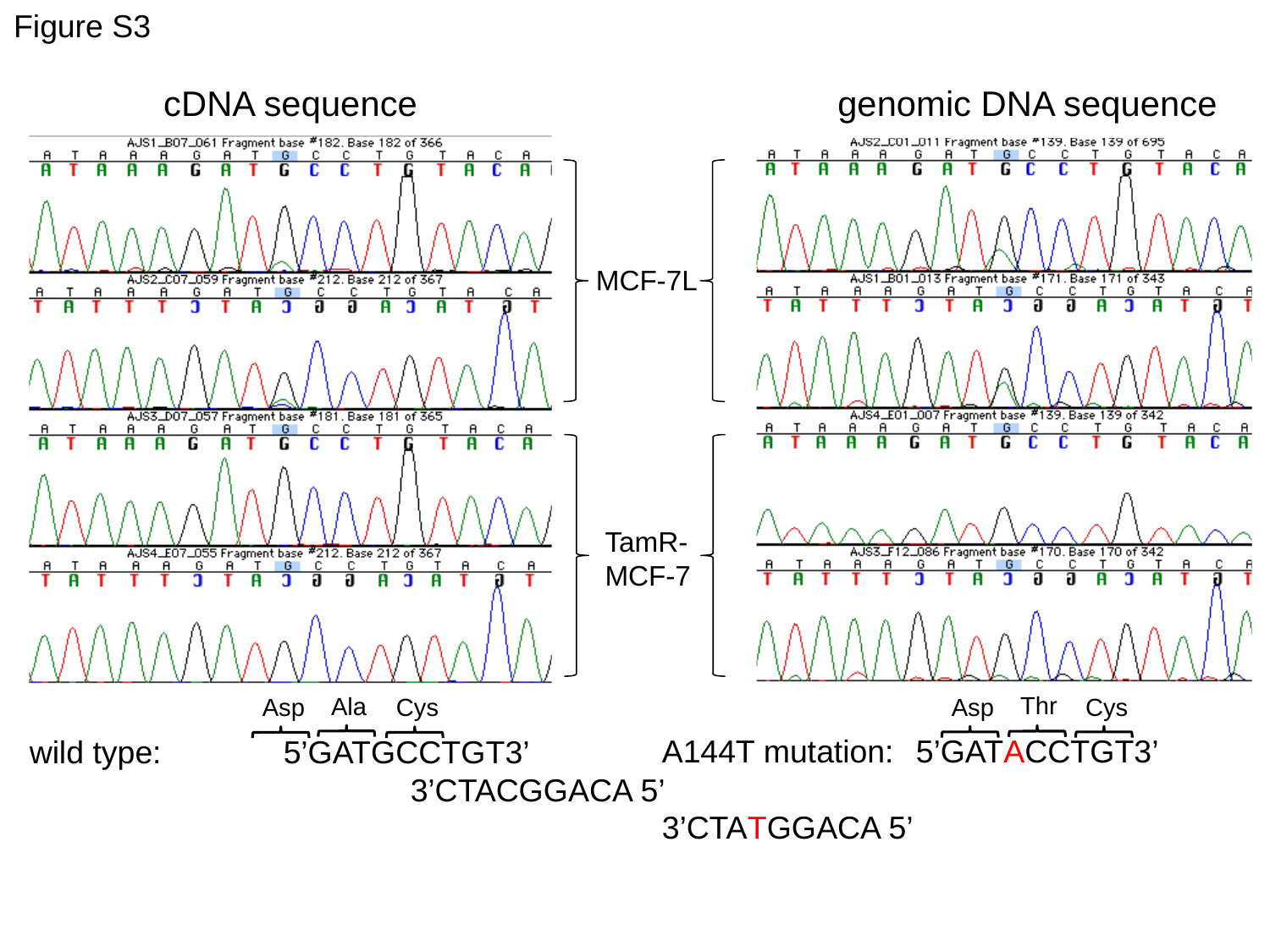

Figure S3
cDNA sequence
genomic DNA sequence
MCF-7L
TamR-MCF-7
Thr
Ala
Asp
Cys
Asp
Cys
A144T mutation: 	5’GATACCTGT3’
				3’CTATGGACA 5’
wild type: 	5’GATGCCTGT3’
			3’CTACGGACA 5’

## Slide 4
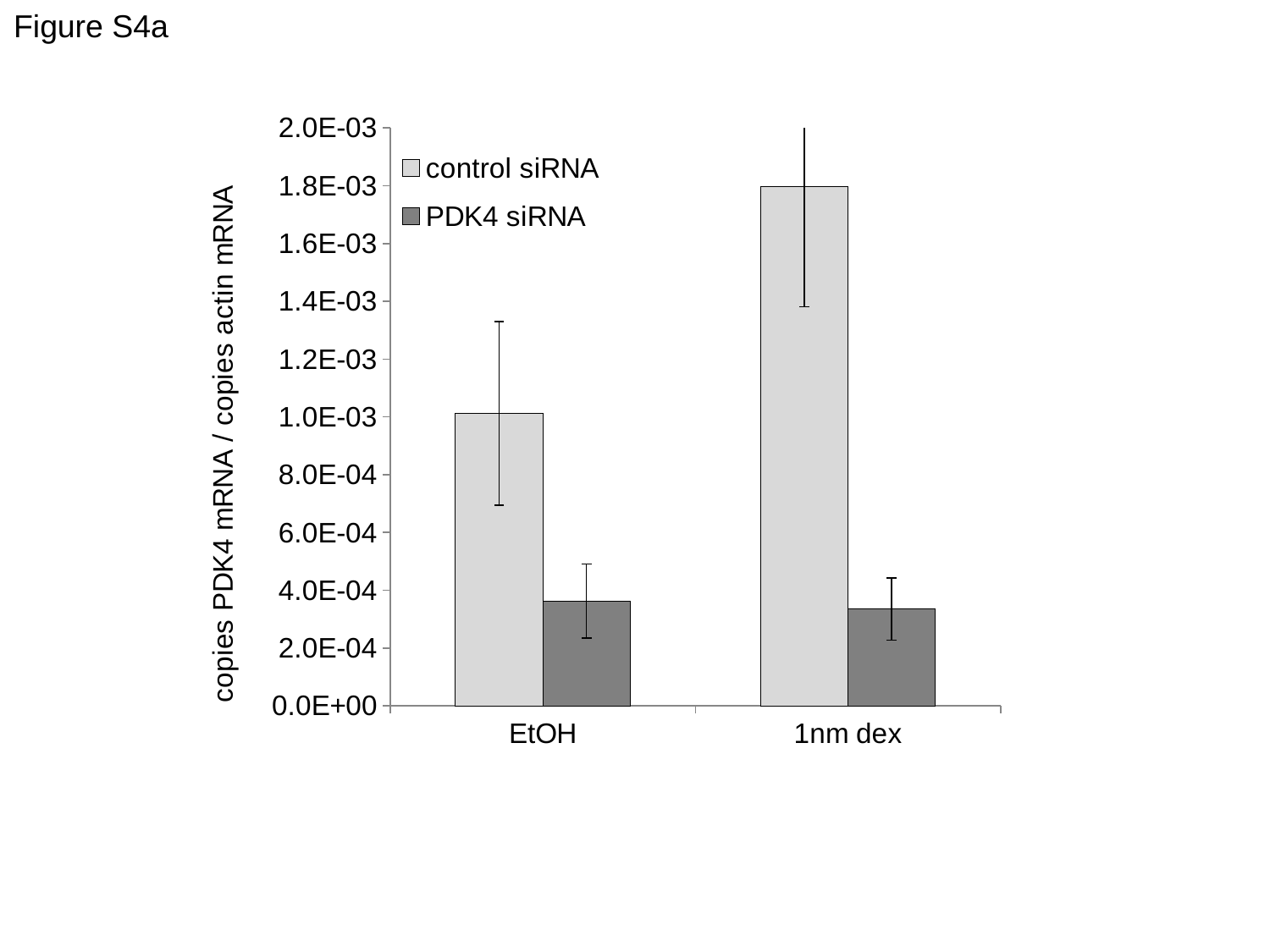

Figure S4a
### Chart
| Category | control siRNA | PDK4 siRNA |
|---|---|---|
| EtOH | 0.0010121114579568 | 0.000362575695546729 |
| 1nm dex | 0.00179635385697146 | 0.000335126930698822 |

## Slide 5
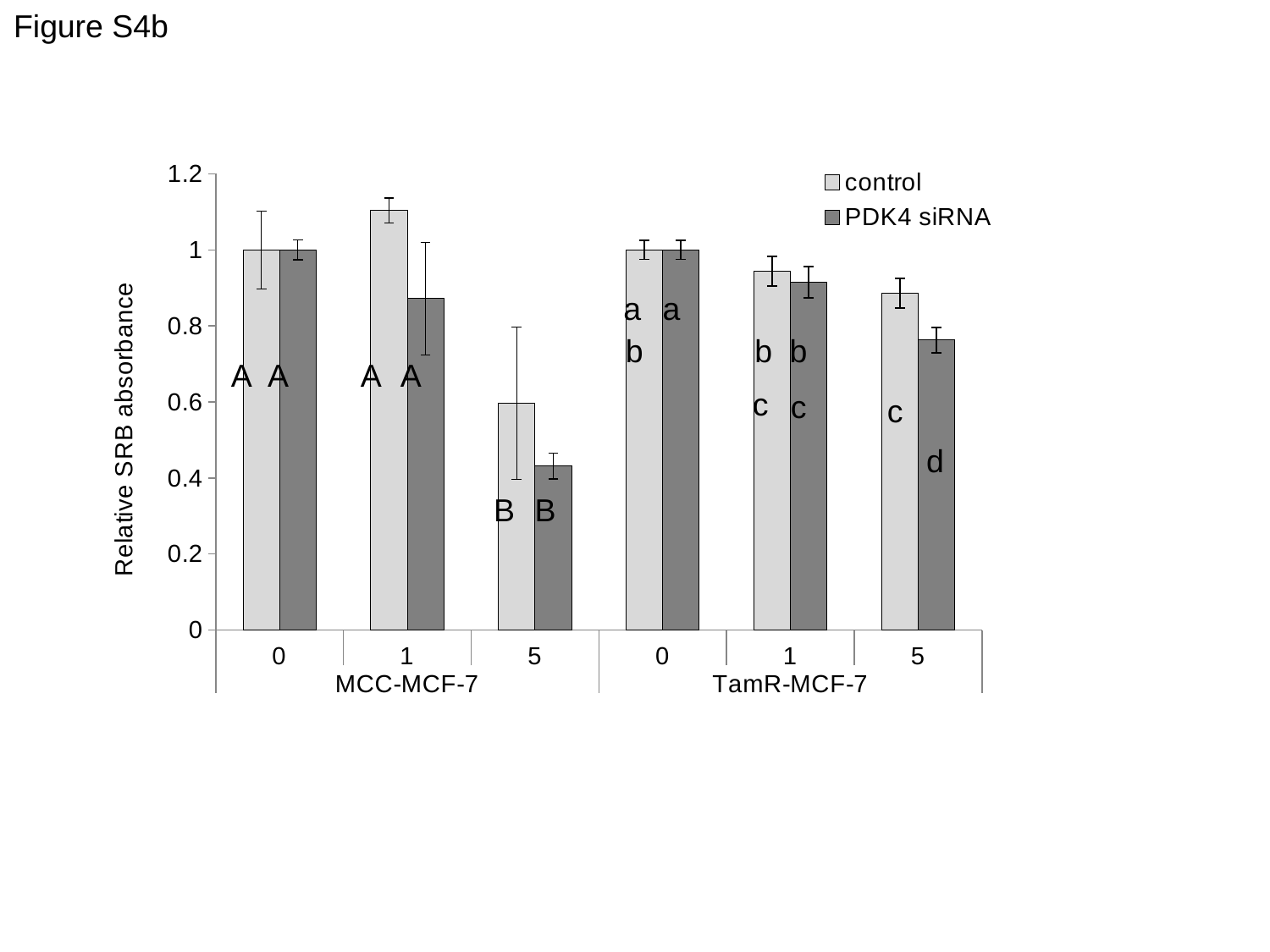

Figure S4b
### Chart
| Category | control | PDK4 siRNA |
|---|---|---|
| 0 | 1.0 | 1.0 |
| 1 | 1.103553777359379 | 0.871785346593502 |
| 5 | 0.596288910084106 | 0.43113238587516 |
| 0 | 1.0 | 1.0 |
| 1 | 0.943601429944595 | 0.915226986451474 |
| 5 | 0.886166042055923 | 0.762598695327141 |a
a
b
b
b
A
A
A
A
c
c
c
d
B
B
